# GENOME-WIDE ASSOCIATION STUDY OF EXTREME LONGEVITY USING WHOLE-GENOME SEQUENCING DATA

**DOI:** 10.1093/geroni/igac059.1556

**Published:** 2022-12-20

**Authors:** Anastasia Gurinovich, Harold Bae, Zeyuan Song, Anastasia Leshchyk, Mengze Li, Stacy Andersen, Thomas Perls, Paola Sebastiani

**Affiliations:** Tufts Medical Center, Boston, Massachusetts, United States; Oregon State University, Corvallis, Oregon, United States; Boston University, Boston, Massachusetts, United States; Boston University, Boston, Massachusetts, United States; Boston University, Boston, Massachusetts, United States; Boston University School of Medicine, Boston, Massachusetts, United States; Boston University, Boston, Massachusetts, United States; Tufts Medical Center, Boston, Massachusetts, United States

## Abstract

Extreme longevity (EL) runs in families which supports the hypothesis that this is a genetically regulated trait. However, with the exception of APOE, genome-wide association studies (GWAS) of EL have not identified many genetic variants that replicate in independent studies. The majority of GWAS of EL have used imputed genotype data. Recently, the Long Life Family Study has generated the largest whole-genome sequencing data of centenarians and matched controls. We perform single-variant and gene-based tests of EL in these data using the nf-gwas-pipeline with the saddle point approximation adjustment of the p-values. These analyses suggest that, in addition to the APOE/TOMM40 region, some uncommon variants of GRM7 (chr3), AUTS2 (chr7), KIF13B (chr8), SLC2A14 (chr12), and ADCY9 (chr16) genes, and other intergenic SNPs in chromosomes 5, 10, and 20 may be implicated with EL.

